# Adiponectin signaling and function in insulin target tissues

**DOI:** 10.1093/jmcb/mjw014

**Published:** 2016-03-30

**Authors:** Hong Ruan, Lily Q. Dong

**Affiliations:** 1 Department of Pharmacology, University of Texas Health Science Center at San Antonio, 7703 Floyd Curl Drive, San Antonio, TX 78229, USA; 2 Department of Cell and Structural Biology, University of Texas Health Science Center at San Antonio, 7703 Floyd Curl Drive, San Antonio, TX 78229, USA

**Keywords:** adiponectin, insulin resistance, cell signaling, APPL1, APPL2, adiponectin receptor

## Abstract

Obesity-linked type 2 diabetes is one of the paramount causes of morbidity and mortality worldwide, posing a major threat on human health, productivity, and quality of life. Despite great progress made towards a better understanding of the molecular basis of diabetes, the available clinical counter-measures against insulin resistance, a defect that is central to obesity-linked type 2 diabetes, remain inadequate. Adiponectin, an abundant adipocyte-secreted factor with a wide-range of biological activities, improves insulin sensitivity in major insulin target tissues, modulates inflammatory responses, and plays a crucial role in the regulation of energy metabolism. However, adiponectin as a promising therapeutic approach has not been thoroughly explored in the context of pharmacological intervention, and extensive efforts are being devoted to gain mechanistic understanding of adiponectin signaling and its regulation, and reveal therapeutic targets. Here, we discuss tissue- and cell-specific functions of adiponectin, with an emphasis on the regulation of adiponectin signaling pathways, and the potential crosstalk between the adiponectin and other signaling pathways involved in metabolic regulation. Understanding better just why and how adiponectin and its downstream effector molecules work will be essential, together with empirical trials, to guide us to therapies that target the root cause(s) of type 2 diabetes and insulin resistance.

## Overview

Adiponectin, also known as Acrp30 (Scherer et al., 1995), AdipoQ (Hu et al., 1996), GBP-28 (Nakano et al., 1996), and apM1 (Maeda et al., 1996), and independently identified by four groups using different approaches (Scherer et al., 1995; Hu et al., 1996; Maeda et al., 1996; Nakano et al., 1996), was originally cloned as an adipocyte-enriched protein highly induced upon 3T3-L1 adipocyte differentiation (Scherer et al., 1995). The human adiponectin gene encodes a 244-amino acid protein of 30 kDa (247 amino acids for the mouse ortholog), whose primary structure includes a signal peptide, a variable region, a collagen-like domain, and a globular domain (Swarbrick and Havel, 2008). The full-length adiponectin protein shares structural similarity with complement factor C1q, tumor necrosis factor-α, and collagens VIII and X. Adipocytes synthesize and secrete multiple forms of adiponectin: low-molecular weight (LMW) trimers (the most basic form), medium-molecular weight (MMW) hexamers, and high-molecular weight (HMW) oligomers of 4–6 trimers (Berg et al., 2002; Pajvani et al., 2003; Waki et al., 2003; Hada et al., 2007). A proteolytic adiponectin fragment, known as globular adiponectin (gAd), also occurs in human plasma (Fruebis et al., 2001; Waki et al., 2005).

During the past 20 years, a large body of work established important roles of adiponectin in metabolic regulation and inflammatory/anti-inflammatory processes. Notably, each adiponectin form appears to have distinct target tissue specificity and modulates unique biological processes (Yamauchi et al., 2002; Tsao et al., 2003). Adiponectin is an insulin sensitizer (Berg et al., 2001; Combs et al., 2001; Yamauchi et al., 2001; Kim and Scherer, 2004), and reduced adiponectin levels (Hotta et al., 2001; Lindsay et al., 2002; Maeda et al., 2002; Spranger et al., 2003; Bajaj et al., 2004) and/or ratios of HMW/LMW (Pajvani et al., 2004; Hara et al., 2006; Lara-Castro et al., 2006) are linked to insulin resistance and metabolic syndrome. When supplied exogenously (Berg et al., 2001; Combs et al., 2001; Yamauchi et al., 2001; Zhao et al., 2015) or overexpressed as a transgene (Combs et al., 2004; Otabe et al., 2007; Wang et al., 2014; Ye et al., 2014), adiponectin suffices to promote insulin action and ameliorates insulin resistance. While adiponectin exerts pro-inflammatory activities in some contexts (Cheng et al., 2012; Fantuzzi, 2013; Wan et al., 2014), it can suppress inflammatory responses (Lovren et al., 2010; Ohashi et al., 2010, 2012; Villarreal-Molina and Antuna-Puente, 2012; Iannitti et al., 2015). Adiponectin enhances the secretion of the anti-inflammatory cytokine IL-10 by cultured human monocyte-derived macrophages and stromal vascular fraction cells prepared from human adipose tissue (Kumada et al., 2004). Intriguingly, adiponectin promotes macrophage polarization toward the anti-inflammatory M2 phenotype (Ohashi et al., 2010). On the other hand, macrophage polarization phenotype regulates the expression of adiponectin receptors (AdipoRs) in ways that classical activation (M1) of macrophages suppresses the expression of AdipoRs, and alternative activation (M2) preserves it (van Stijn et al., 2015). Remarkably, adiponectin elicits antagonistic responses in the two macrophage-polarization phenotypes. In M1 macrophages, adiponectin induced the expression of pro-inflammatory cytokines including TNF-α, IL-6, and IL-12, as well as AdipoRs. In M2 macrophages, adiponectin triggered the expression of the anti-inflammatory cytokine IL-10 without affecting AdipoR levels (van Stijn et al., 2015).

Recent studies have also begun to reveal mechanisms of adiponectin actions and the cellular circuitry downstream of the adiponectin receptors. While these advances offer novel opportunities for diabetes treatment, multiple considerations limit the development of adiponectin as a pharmacological agent in a clinical setting. First, under physiological conditions, the circulating plasma concentrations of adiponectin in humans range from 2 to 20 µg/ml (Turer and Scherer, 2012), more than 1000-fold higher than other hormonal regulators such as insulin. This abundance would make its development for clinical use unlikely. Second, adiponectin circulates in multiple forms of oligomers, each with its unique cellular target(s) and signaling pathways (Tsao et al., 2003). Currently, selective enrichment of a particular multimeric form of adiponectin *in vivo* remains a challenge. Lastly, various forms of adiponectin have relatively short half-life: 32 min for trimers and 83 min for HMW and MMW proteins (Halberg et al., 2009). Conceptually, these characteristics necessitate multiple high doses of adiponectin if used as a therapeutic agent, a measure with potentially high clinical risks. Thus, understanding the mechanistic details of adiponectin signal transduction could reveal new opportunities for clinical treatment, tailored to its underlying biology and pathophysiology.

Here, we consider five aspects of adiponectin action and signal transduction with the potential for drug development: (i) tissue-specific functions of adiponectin; (ii) adiponectin receptors AdipoR1, AdipoR2, and T-cadherin; (iii) adiponectin receptor signaling; (iv) adiponectin signaling pathway crosstalks with other pathways involved in metabolic regulation; and (v) adipoR-independent pathways. Our goal is not to comprehensively review these areas, but rather, to identify recent advancements and updates in adiponectin biology and explore the therapeutic potential of targeting adiponectin signal transduction.

## Tissue-specific functions of adiponectin

Insulin resistance, a defining feature of type 2 diabetes, is a state in which physiological concentrations of insulin produce a less than normal response, thereby impairing the capacity of insulin targets to address the metabolic needs of the body. Clinically, this presents as hyperinsulinemia, dyslipidemia, hyperglycemia, elevated circulating inflammatory markers, and diminished plasma adiponectin levels, with increased morbidity and mortality due to cardio- and cerebro-vascular diseases or kidney and liver dysfunction and failure (Padmalayam and Suto, 2013; Grundy, 2015; Taskinen and Boren, 2015). Several pathways contribute to the etiology of insulin resistance at a cellular level, including defective insulin signal transduction, impaired effector molecules within insulin-dependent pathways, and enhanced insulin-antagonizing pathways (Boucher et al., 2014).

**Figure 1:**
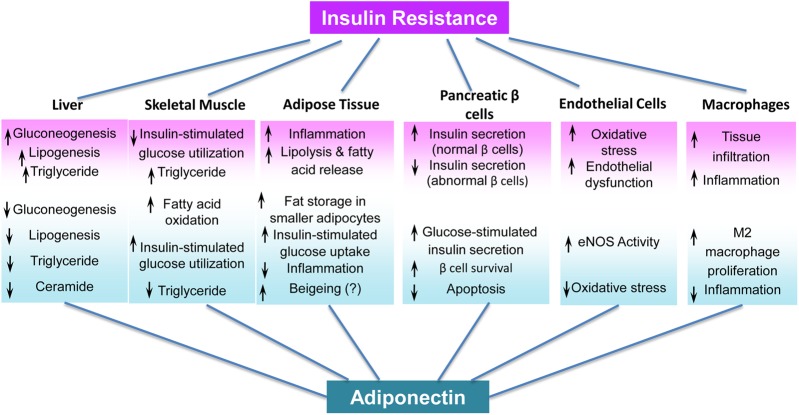
Summary of tissue-specific functions of adiponectin.

Adiponectin is a widely recognized insulin sensitizer, and several approaches have identified and characterized its insulin-sensitizing activities, *in vivo* target tissues, and underlying mechanisms (Figure 1). First, intraperitoneal injection of full-length adiponectin reduces plasma glucose levels in mice, an effect from suppressed hepatic glucose production that is independent of insulin levels or glucose disposal rate at peripheral tissues (Berg et al., 2001). Second, adiponectin inhibits the expression of phosphoenolpyruvate carboxykinase and glucose-6-phosphatase (Yamauchi et al., 2002), thereby suppressing gluconeogenesis. Thus, the liver is a major target tissue of full-length adiponectin. Supporting this notion, increasing HMW adiponectin via fat-specific overexpression of DsbA-L, an adipose-abundant protein that promotes adiponectin multimerization, stimulates hepatic AMPK-α phosphorylation at Thr^172^ that is essential for 5′-AMP-activated protein kinase (AMPK) activation, and ameliorates high-fat diet-induced insulin resistance and hepatosteatosis (Liu et al., 2008, 2012), without affecting AMPK activity in skeletal muscle (Liu et al., 2012). Another independent study showed that adiponectin administration increases fatty acid oxidation in skeletal muscle, and suppresses lipid accumulation in the liver by activating AMPK, thereby reducing triglyceride content in the liver and muscle and improving overall *in vivo* insulin sensitivity (Yamauchi et al., 2001, 2002). It has also been suggested that each oligomeric form of adiponectin has specific tissue targets. The globular form of adiponectin stimulates the AMPK pathway in skeletal muscle, resulting in increased fatty acid oxidation and glucose utilization (Tomas et al., 2002; Yamauchi et al., 2002). These studies establish skeletal muscle as another major adiponectin target.

The current body of work has implicated adiponectin in a spectrum of tissue-specific activities (Figure 1). Adiponectin targets tissue-macrophages and indirectly regulates insulin sensitivity by modulating immune responses. Adiponectin suppresses inflammatory responses (Lovren et al., 2010; Ohashi et al., 2010, 2012; Villarreal-Molina and Antuna-Puente, 2012; Iannitti et al., 2015) and promotes macrophage polarization toward the anti-inflammatory M2 phenotype (Ohashi et al., 2010). Meanwhile in some physiological and pathophysiological contexts, adiponectin has also been shown to induce pro-inflammatory activities (Cheng et al., 2012; Fantuzzi, 2013; Wan et al., 2014). A variety of factors, including experimental conditions, adiponectin quality, and the short-term memories of immune and non-immune cells, may underlie the reported discrepancies, which were discussed in detail in another review (Esmaili et al., 2014). Notably, adiponectin exerts anti-apoptotic effects in cardiac myocytes (Holland et al., 2011) and pancreatic β-cells (Brown et al., 2010; Wijesekara et al., 2010), and mitigates oxidative stress in endothelial cells (Wong et al., 2011) and podocytes (Sharma, 2009). Despite these well-established endocrine effects of adiponectin, its autocrine/paracrine effects remain elusive. For example, adiponectin lowers hepatic ceramide content through enhanced ceramide catabolism and production of an anti-apoptotic metabolite, sphingosine-1-phosphate (S1P), thereby improving insulin sensitivity, suppressing inflammation, and promoting survival (Holland et al., 2011). Nevertheless, the role of adiponectin in the control of adipose ceramide content is unclear. Adiponectin overexpression in the adipose tissue of *ob/ob* mice reduces adipose tissue and systemic inflammation, and promotes fat storage in subcutaneous fat depots comprising smaller adipocytes, resulting in improved systemic insulin sensitivity and pancreatic β-cell survival (Kim et al., 2007). However, the physiological effects of adipocyte-derived endogenous adiponectin on adipose tissue are not known.

Although the peripheral target tissues of adiponectin are known, its roles in the central nervous system, particularly its effect on feeding behavior, are controversial (Kubota et al., 2007; Coope et al., 2008; Bassi et al., 2012). Perhaps the most vague aspect of adiponectin action is its effect on thermogenesis. One study showed that adiponectin inhibits thermogenesis in an adiponectin receptor-independent manner (Qiao et al., 2014), while another revealed that adiponectin promotes cold exposure-induced subcutaneous white adipose tissue beigeing and thermogenesis by promoting M2 macrophage proliferation (Hui et al., 2015). Further studies, including clinical trials, are needed to identify and characterize the roles of adiponectin in the regulation of beigeing and thermogenesis.

## Adiponectin receptors: AdipoR1, AdipoR2, and T-cadherin

Adiponectin receptors, AdipoR1 and AdipoR2, were originally identified by screening a skeletal muscle expression library for cDNAs whose encoded proteins bind to the globular domain of adiponectin (Yamauchi et al., 2003a). AdipoR1 and AdipoR2 regulate metabolic gene expression and insulin sensitivity in insulin target tissues, and are important in the pathophysiology of insulin resistance and diabetes (Yamauchi et al., 2007, 2014). Both receptors contain seven-transmembrane domains and belong to the PAQR family, which has the opposite transmembrane topology to the G-protein coupled receptors, and have N-terminus in the cytoplasm and C-terminus facing extracellular space (Tang et al., 2005; Tanabe et al., 2015). Our laboratory independently identified AdipoR1 as an adiponectin receptor using a yeast two-hybrid system (Mao et al., 2006). Using full-length adiponectin, we mapped the ligand-binding site on AdipoR1 to be the C-terminal region, further corroborating its unique transmembrane topology (Mao et al., 2006).

A recent crystal structure study of the human AdipoR1 and AdipoR2 has provided mechanistic insights into their functions (Tanabe et al., 2015). The most provocative aspect of their structures is a large cavity enclosed by the seven-transmembrane helices in both AdipoR1 and AdipoR2, containing three conserved histidine residues coordinated to a zinc ion (Tanabe et al., 2015). The zinc-binding motif has been implicated in adiponectin-stimulated activation of AMPK and PPAR-α (Tanabe et al., 2015). These studies point to new avenues by which targeted adiponectin signaling can improve insulin sensitivity.

Although ubiquitously expressed, AdipoR1 is most abundant in skeletal muscle; AdipoR2 expression, however, is mostly restricted in the liver (Yamauchi et al., 2003a). Both receptors can elicit a series of downstream signaling events. Overexpression of AdipoR1 in the liver activates AMPK, suppresses hepatic gluconeogenesis and *de novo* lipogenesis, and promotes fatty acid oxidation (Yamauchi et al., 2007). AdipoR1 ablation impairs adiponectin-mediated activation of AMPK and SIRT1, producing an insulin-resistant state (Iwabu et al., 2010). At present, however, the function of AdipoR2 has been debated. Yamauchi et al. (2007) reported that the AdipoR2-null mice exhibit similar phenotypes as mice lacking AdipoR1. Independently, two groups showed that AdipoR2-deficient mice are resistant to high-fat diet-induced obesity and dyslipidemia and exhibit markedly improved glucose tolerance, insulin sensitivity, physical activity, and energy expenditure: phenotypes that are the opposite to those observed in the AdipoR1 knockout mice (Bjursell et al., 2007; Liu et al., 2007). Thus, the exact functional roles of AdipoR2 require further investigation.

In addition to AdipoR1 and AdipoR2, T-cadherin has been identified as a receptor for the HWM form of adiponectin, but not for trimeric or globular adiponectin (Hug et al., 2004). Lacking the intracellular structural domain, T-cadherin has been postulated as the binding protein for adiponectin and plays a key role in adiponectin signaling (Hebbard et al., 2008; Denzel et al., 2010; Parker-Duffen et al., 2013; Matsuda et al., 2015). Circulating levels of adiponectin, particularly HMW form of adiponectin, are elevated in T-cadherin-deficient mice (Denzel et al., 2010). Despite these earlier reports, the function of T-cadherin in adiponectin signaling, particularly its relationships to AdipoR1 and AdipoR2, remains to be characterized. Given that the HMW adiponectin is the more active and effective insulin sensitizer, future research efforts should elucidate the molecular mechanisms by which HMW adiponectin functions in its target cells.

## Adiponectin receptor signaling

### APPL1 protein

**Figure 2:**
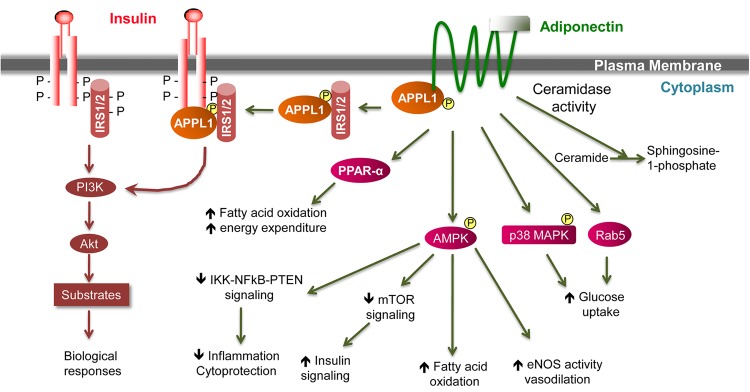
Schematic representation of adiponectin signal transduction pathway implicating a crosstalk with the insulin signaling pathway. Activation of insulin and adiponectin receptors by their respective ligands triggers a cascade of signaling events. Most of the metabolic effects of insulin are mediated by the PI3K/AKT pathway, leading to biological responses that include increased protein synthesis, lipogenesis, glucose uptake and utilization, and glycogen synthesis, and reduced lipolysis and gluconeogenesis. In the case of adiponectin, APPL1 interacts with AdipoR1 or AdipoR2 through its C-terminal PTB and CC domains, and mediates the effects of adiponectin on the activation of multiple pathways including PPAR-α, AMPK, and p38 MAPK. Both AdipoR1 and AdipoR2 are associated with ceramidase activities that are activated upon adiponectin binding. One key binding partner of IRS1/2 is APPL1, which promotes IRS1/2 binding to the insulin receptor and enhances insulin signaling transduction. This crosstalk between insulin and adiponectin signaling pathways is a major mechanism by which adiponectin sensitizes insulin action in insulin target tissues.

Adiponectin elicits a number of downstream signaling events. However, no intrinsic protein kinase activity or phosphorylation in response to adiponectin has ever been detected in either AdipoR1 or AdipoR2. Furthermore, replacement of the tyrosine residues within the intracellular N-terminus by site-directed mutagenesis has no impact on adiponectin signaling (Mao et al., 2006). Thus, the adiponectin receptors are likely transmembrane receptors that undergo conformational change and couple the intracellular domain with other signaling molecules upon extracellular adiponectin binding. Using yeast two-hybrid technology, our laboratory identified APPL1 as the intracellular binding partner of AdipoR1 and AdipoR2 (Mao et al., 2006). The human *Appl1* gene is located in the 3p14.3-21.1 region and encodes a 709-amino acid protein of 78 kDa. Human genetic studies suggest that SNPs and point mutations in the *Appl1* coding region correlate with body fat distribution and a high prevalence of diabetes (Fang et al., 2008; Prudente et al., 2015). APPL1 is highly hydrophilic with no potential transmembrane region and consisted of multiple structural and functional domains including BAR, PH, PTB, and coiled coil (CC) (Mitsuuchi et al., 1999; Liu et al., 2002). APPL1 directly binds to the intracellular domains of AdipoR1 and AdipoR2 via its C-terminal PTB and CC domains, thereby mediating the actions of adiponectin in the regulation of energy metabolism and insulin sensitivity (Figure 2). In cultured skeletal muscle cells, suppression of APPL1 expression diminished adiponectin-induced glucose uptake and GLUT4 translocation (Mao et al., 2006). On the other hand, overexpression of APPL1 enhanced the stimulatory actions of adiponectin in glucose metabolism in muscle (Mao et al., 2006). Rab5, a GTPase downstream of APPL1, plays an important role in APPL1-mediated adiponectin signaling (Mao et al., 2006).

The major action of adiponectin on lipid metabolism is to promote fatty acid oxidation, a process in which AMPK and acetyl CoA carboxylase (ACC) play a critical role. Research in our laboratory revealed the significance and detailed mechanisms by which APPL1-mediated signaling activates AMPK (Zhou et al., 2009; Deepa et al., 2011). Upon adiponectin stimulation, APPL1 binds to protein phosphatase 2A (PP2A) and protein kinase Cζ (PKCζ), thereby activating PP2A and inactivating PKCζ via dephosphorylation. The inactivation of PKCζ results in the dephosphorylation of Ser^307^ on liver kinase B1 (LKB1), allowing LKB1 to translocate from nucleus to cytoplasm and activate AMPK (Deepa et al., 2011). In addition to the AMPK pathway, we further demonstrated that APPL1 also mediates adiponectin-induced activation of the p38 MAPK pathway (Mao et al., 2006) and investigated its impact on the anti-inflammatory actions of adiponectin (Xin et al., 2011). We show that upon induction of adiponectin, APPL1 tethers p38 MAPK together with its upstream activating kinases including transforming growth factor-β activated kinase 1 (TAK1) and mitogen-activated protein kinase kinase 3 (MKK3), thereby expediting the phosphorylation of key enzymes of this pathway (Xin et al., 2011). Interestingly, the action of APPL1 on p38 MAPK pathway is specific to adiponectin, whereas its impact on TNF-α-induced p38 MAPK activation is limited (Xin et al., 2011). We further demonstrated the *in vivo* involvement of APPL1 in mediating the actions of adiponectin. Whole-body knockout of APPL1 impairs adiponectin signaling and results in insulin resistance in major insulin target tissues (Ryu et al., 2014).

Subsequent studies by several laboratories further corroborated that APPL1 functions downstream of adiponectin in various tissues and cell types. Cheng et al. (2007) reported that in cultured HUVEC cells, APPL1 binds to the cytoplasmic tails of AdipoR1 and AdipoR2 in response to adiponectin, thereby activating AMPK and eNOS and resulting in the production of NO. APPL1 overexpression potentiates insulin-stimulated AKT signaling and suppresses hepatic gluconeogenesis, whereas knocking down APPL1 attenuates insulin-stimulated AKT phosphorylation (Cheng et al., 2009). Chandrasekar et al. (2008) showed that adiponectin treatment blocks IL-18-induced endothelial cell death by activating AMPK, which inhibits IKK/NF-κB/PTEN-triggered apoptosis. Fang et al. (2010) reported that binding of APPL1 to AdipoR1 mediates adiponectin-induced AMPK activation in cardiac myocytes. Coope et al. (2008) demonstrated that injection of adiponectin into the third ventricle triggers interactions of APPL1 with AdipoR1 and AdipoR2, and that the insulin- and leptin-sensitizing actions of adiponectin are mediated through AdipoR1 but not AdipoR2.

### APPL2 protein

APPL2 is an isoform of APPL1 and shares 54% homology in amino acid sequence with APPL1 protein (Miaczynska et al., 2004; Wang et al., 2009). Similar to APPL1, APPL2 has an N-terminal BAR domain, central PH domain, and C-terminal PTB domain. APPL2 mediates FSH signal transduction by binding to APPL1 via their respective BAR domains, which results in the formation of the FSH receptor–APPL1–AKT2 complex (Nechamen et al., 2007). Notably, APPL2 does not directly interact with AKT2 (Nechamen et al., 2007; Chial et al., 2008). Research in our laboratory revealed that APPL2 negatively modulates adiponectin signaling in skeletal muscle cells (Wang et al., 2009). APPL2 directly binds to AdipoR1 or AdipoR2 via its BAR domain, thereby preventing the interaction of APPL1 with AdipoRs. Thus, APPL2 blocks adiponectin signaling through AdipoR1 and AdipoR2 by competitive inhibition of APPL1. In addition, APPL2 heterodimerizes with APPL1, thereby reducing the binding of APPL1 to AdipoRs and impairing the actions of adiponectin. Interestingly, adiponectin modulates the dissociation of the APPL1/APPL2 heterodimers, which can also be triggered by insulin and metformin (Wang et al., 2009). Because APPL1 and APPL2 exert opposite actions in mediating adiponectin signaling, we originally proposed the ‘Yin and Yang’ modulatory concept (Wang et al., 2009).

Adiponectin circulates at a high concentration without major fluctuations (Merl et al., 2005). In moving forward, it is important to address the following questions: (i) Does adiponectin bind to its receptors continuously? (ii) If the binding is relatively continuous, the intracellular regulatory mechanisms become the key checkpoints in modulating adiponectin signaling transduction and action. Then what are the regulatory mechanisms? The Yin and Yang modulatory concept involving APPL1/APPL2 offers a detailed molecular mechanism by which adiponectin regulates lipid and carbohydrate metabolism. Consistent with the Yin-Yang theory, mice with muscle-specific APPL2 ablation or overexpression show respective enhanced or impaired insulin sensitivity, insulin-stimulated GLUT4 translocation, and glucose uptake in skeletal muscle (Cheng et al., 2014). The opposing roles of APPL1 and APPL2 were also observed in the regulation of the PI3K/AKT/NF-κB pathway in macrophages (Mao et al., 2014).

### The AMPK pathway

AMPK, a protein kinase regulated by AMP, is a widely recognized cellular sensor for metabolic state. In skeletal muscle, both full-length adiponectin and the globular domain have been shown to trigger AMPK phosphorylation, leading to AMPK activation (Tomas et al., 2002; Wu et al., 2003). We found that APPL1 plays a key role in adiponectin-mediated AMPK phosphorylation (Mao et al., 2006; Zhou et al., 2009; Deepa et al., 2011). In primary culture of skeletal muscle isolated from obese individuals or patients with type 2 diabetes, AMPK phosphorylation in response to adiponectin is greatly reduced, demonstrating that impaired signaling downstream of the adiponectin receptor may impair the actions of adiponectin or cause adiponectin resistance (Chen et al., 2005). Adiponectin has also been shown to induce AMPK phosphorylation in the liver (Yamauchi et al., 2002). Notably, recent reports suggest a limited role of the AMPK pathway in the regulation of gluconeogenesis (Miller et al., 2011). At present, however, no other mechanisms, i.e. non-AMPK pathways, have been shown to mediate the effects of adiponectin on gluconeogenesis in the liver.

### The PPAR pathway

Another key transcription factor in metabolic regulation is PPAR-α. In skeletal muscle, adiponectin drastically increases the expression and activity of PPAR-α, which in turn upregulates acetyl CoA oxidase (ACO) and uncoupling proteins (UCPs), thereby promoting fatty acid oxidation and energy expenditure (Yamauchi et al., 2003a). In the liver, adiponectin upregulates several PPAR-α target genes including CD36, which modulates hepatic fatty acid uptake and metabolism (Yamauchi et al., 2001), and ACO, which regulates fatty acid oxidation (Yamauchi et al., 2001). In addition, adiponectin has been shown to increase hepatic glucose uptake via PPAR-α, thereby improving hepatic insulin sensitivity (Yamauchi and Kadowaki, 2013).

Thiazolidinedione (TZD) class of PPAR-γ ligands upregulates adiponectin expression in adipocytes (Maeda et al., 2001; Combs et al., 2002). The effect of TZDs on improving glucose tolerance is impaired in adiponectin-deficient mice, indicating that adiponectin mediates, at least in part, the insulin-sensitizing actions of TZDs (Nawrocki et al., 2006). The expression of PPAR-γ is markedly increased in 3T3-L1 cells overexpressing adiponectin, which is associated with enhanced adipocyte differentiation (Fu et al., 2005), indicating that adiponectin promotes the PPAR-γ pathway, thereby activating a positive feed-back loop that increases adiponectin expression and adipocyte differentiation.

### Other adiponectin signaling pathways

In addition to the pathways discussed above, adiponectin has been shown to induce calcium release from sarcoplasmic reticulum in myocytes or promote calcium influx, thereby activating Ca^2+^/calmodulin-dependent protein kinase kinase (CaMKK-β) and AMPK, which results in activation of SIRT1 and PPAR-α and increase of mitochondria biogenesis (Zhou et al., 2009; Iwabu et al., 2010). In addition, ceramide-mediated pathways also have been implicated in mediating the actions of adiponectin. Holland et al. (2011) reported that both AdipoR1 and AdipoR2 are associated with ceramidase activities, which, upon adiponectin binding, potently enhances ceramides conversion to S1P, a process that is independent of AMPK. Administration of recombinant adiponectin in leptin-deficient *Lep*^ob/ob^ mice markedly reduced hepatic ceramide content, which is associated with increased insulin sensitivity (Holland et al., 2011). The p38 MAPK pathway also plays a role in adiponectin signaling (Mao et al., 2006; Xin et al., 2011). Thus, multiple cellular pathways likely mediate the pleiotropic effects of adiponectin in various insulin target tissues, depending on contexts (Figure 2).

## Cross talks with the insulin signaling pathway

Several groups independently reported the insulin-sensitizing actions of adiponectin in 2001 (Berg et al., 2001; Combs et al., 2001; Fruebis et al., 2001). Overexpression or administering recombinant adiponectin reduces blood glucose levels and ameliorates insulin resistance in obese mice, which are independent of plasma insulin levels (Berg et al., 2001; Combs et al., 2001; Fruebis et al., 2001; Yamauchi et al., 2003b). Conversely, ablation of the adiponectin gene exacerbates insulin resistance and hyperglycemia in mice fed on a high-fat diet (Kubota et al., 2002; Maeda et al., 2002; Nawrocki et al., 2006). Research in our laboratory focused on the mechanisms underlying the insulin-sensitizing actions of adiponectin. We show that in skeletal muscle, adiponectin promotes tyrosine phosphorylation of IRS1 and AKT phosphorylation via inhibiting p70 S6K phosphorylation and serine phosphorylation of IRS1, thereby increasing insulin sensitivity (Wang et al., 2007). In parallel, adiponectin activates the LKB1/AMPK/TSC1/2 pathway, which blocks the inhibitory actions of the mTOR/p70 S6K pathway on insulin signaling (Wang et al., 2007). Interestingly, APPL1 promotes IRS1/2 binding to the insulin receptor (IR) (Ryu et al., 2014). Under basal conditions, APPL1 forms a complex with IRS1/2; the presence of insulin or adiponectin triggers phosphorylation of Ser^401^ on APPL1, which brings APPL1 to the IR, forming the IR–APPL1–IRS1/2 complex (Figure 2). This process plays a key role in improving insulin sensitivity (Ryu et al., 2014). Using APPL1 knockout mice, we find that APPL1 sensitizes insulin actions via modulating IRS1/2 but not tyrosine phosphorylation of the IR, further corroborating the mechanisms of APPL1 action. Notably, recent studies on the *in vivo* effects of APPL2 on insulin signaling also demonstrated the ‘Yin-Yang’ modulatory actions of APPL1 and APPL2 (Cheng et al., 2014). Yet, APPL2 does not seem to directly regulate the interactions between IRS1/2 and the IR (Ryu et al., 2014). Thus, APPL1 plays a key role in the crosstalk between adiponectin and insulin signaling pathways. Elucidating the mechanisms underlying the insulin-sensitizing actions of adiponectin will provide a guide to our understanding and treatment of insulin resistance (Figure 2).

## AdipoR-independent actions of adiponectin

Despite convincing experimental data about adiponectin receptor-mediated actions in various adiponectin target tissues, emerging reports reveal that adiponectin exerts some receptor-independent activities. Certain adiponectin-responsive pre-autonomic (PA) neurons in the hypothalamus do not express AdipoR1 or AdipoR2 (Hoyda et al., 2009). Several hypothetical models are consistent with the data. Adiponectin could act indirectly on those AdipoR^−^ PA neurons through endocannabinoids or neuromodulatory peptides released from the AdipoR^+^ neurons in response to adiponectin. Another distinct possibility is that adiponectin interacts directly with a yet to be identified AdipoR in those PA neurons. Supporting this, Takemura et al. (2007) demonstrated that adiponectin facilitates the uptake of early apoptotic cells by macrophages and modulates inflammatory responses through a receptor-dependent pathway that involves calreticulin. RNAi-mediated knocking down of AdipoR1, AdipoR2, and T-cadherin does not affect adiponectin-stimulated removal of apoptotic bodies by macrophages via calreticulin (Takemura et al., 2007), indicating that the process is independent of the known AdipoRs involved in the classical metabolic regulation.

Conceptually, receptor-dependent intracellular signaling usually requires very low circulating ligand concentrations, whereas receptor-independent effects are likely mediated by much higher ligand concentrations. The physiological concentrations of plasma adiponectin is ∼1000-fold higher than that of insulin (Turer and Scherer, 2012), and further studies are needed to determine whether adiponectin functions through both receptor-dependent and receptor-independent mechanisms.

## Future challenges and new horizons

While adiponectin itself is not a suitable candidate for insulin-sensitizing drugs, components of the adiponectin signaling pathway are promising druggable targets. Recent studies show that a small-molecule activator of the adiponectin receptor, AdipoRon, improves glucose tolerance and ameliorates insulin resistance in mice fed a high-fat diet (Okada-Iwabu et al., 2013); furthermore, AdipoRon improves metabolic parameters and prolongs life span in *db/db* mice, a genetic mouse model for diabetes (Okada-Iwabu et al., 2013). Thus, small molecules that enhance adiponectin signaling may be viable options for the treatment of obesity-linked metabolic diseases including type 2 diabetes. However, the current challenge is that the K_d_ for small molecules such as AdipoRon binding to the AdipoR1 or AdipoR2 is much higher than that of the full-length or globular domain of adiponectin, indicating that high-affinity small-molecule AdipoR activators remain to be identified.

Adiponectin circulates at a high and constant concentration; how the adiponectin signaling pathway is regulated is another unsettled question. As such, can we target key molecular mediators in the adiponectin signaling pathway such as APPL1, APPL2, or protein kinases that modify APPL1/2 activity to improve insulin sensitivity? Certain metabolic diseases, such as type 1 diabetes (Maahs et al., 2007; Leth et al., 2008) and IR antibody-induced type B insulin resistance (Semple et al., 2007), exhibit high plasma concentrations of adiponectin. Whether this phenomenon is a result of adiponectin resistance, or compensatory increase to counteract the deficient or impaired insulin signaling, is yet to be determined. Thorough understanding of adiponectin and its downstream signaling pathways will provide a guide for the development of novel drugs in the treatment of obesity-related metabolic diseases.

## Funding

This work was supported by grants from the National Institutes of Health (R01 DK102965) and the American Diabetes Association (#7-13-BS-043) to L.Q.D.

**Conflict of interest:** none declared.
